# Intracranial Stenting as Rescue Therapy After Failure of Mechanical Thrombectomy for Basilar Artery Occlusion: Data From the ANGEL-ACT Registry

**DOI:** 10.3389/fneur.2021.739213

**Published:** 2021-09-30

**Authors:** Gang Luo, Feng Gao, Xuelei Zhang, Baixue Jia, Xiaochuan Huo, Raynald Liu, Man Sum Chi, Gaoting Ma, Guangge Peng, Jingyu Zhang, Zhongqi Qi, Xu Guo, Bin Han, Xu Tong, Bo Wang, Ligang Song, Lian Liu, Zijun He, Dapeng Mo, Ning Ma, Xuan Sun, Ming Yang, Zhongrong Miao

**Affiliations:** ^1^Interventional Neuroradiology Center, Beijing Tiantan Hospital, Capital Medical University, Beijing, China; ^2^China National Clinical Research Center for Neurological Diseases, Beijing, China; ^3^Beijing Institute of Brain Disorders, Capital Medical University, Beijing, China; ^4^Department of Medicine and Geriatrics, Tuen Mun Hospital, Tuen Mun, Hong Kong, SAR China; ^5^Department of Neurology, Shanxi Provincial People's Hospital, Taiyuan, China; ^6^Department of Interventional Neurology, Beijing Anzhen Hospital, Capital Medical University, Beijing, China

**Keywords:** basilar artery occlusion, mechanical thrombectomy, rescue stenting, posterior circulation, acute stroke, recanalization

## Abstract

**Background and Purpose:** Studies on rescue therapy for acute posterior circulation stroke due to basilar artery occlusion (BAO) are limited in the modern era of mechanical thrombectomy (MT). The aim of this study was to evaluate the safety and efficacy of rescue stenting (RS) following MT failure in patients with BAO.

**Methods:** Data were collected from the Endovascular Treatment Key Technique and Emergency Work Flow Improvement of Acute Ischemic Stroke (ANGEL-ACT) prospective registry in China. Patients who underwent MT for BAO with failure of recanalization were enrolled in this study. The patients were divided into the RS and non-RS groups. Clinical and laboratory findings, procedural details, and clinical outcomes were compared between the two groups.

**Results:** Overall, 93 patients with acute BAO were analyzed. The RS group included 81 (87.1%) patients, and the non-RS group included 12 patients. A modified treatment in cerebral infarction (mTICI) score of 2b/3 was achieved in 75 (92.6%) patients in the RS group. Compared with the non-RS group, the RS group had a significantly higher rate of successful recanalization and favorable clinical outcomes (modified Rankin Scale score at 90 days post-procedure, 0–3: 16.7 vs. 51.9%, respectively; *P* = 0.023) without an increase in the rate of symptomatic intracranial hemorrhage and a significantly lower mortality rate (58.3 vs. 18.5%, respectively; *P* = 0.006). Furthermore, the use of a glycoprotein IIb/IIIa inhibitor improved the rate of recanalization of the target artery without increasing the rate of symptomatic intracranial hemorrhage.

**Conclusions:** Permanent stenting appears to be a feasible rescue modality when MT fails and might provide functional benefits in patients with acute ischemic stroke due to BAO.

## Introduction

The efficacy and safety of mechanical thrombectomy (MT) in acute anterior circulation stroke have been well-documented in both clinical trials and observational studies (1–8). MT has become the mainstay treatment for acute ischemic stroke caused by large vessel occlusion of the proximal anterior circulation; however, there is no consensus regarding the treatment of acute posterior circulation stroke. Recently, the results of the Endovascular Interventions vs. Standard Medical Treatment Trial (BEST) trial and registry study (EVT for Acute Basilar Artery Occlusion Study) (BASILAR) demonstrated that patients in whom successful reperfusion [modified treatment in cerebral infarction (mTICI) score of 2b or 3] was achieved had significantly more favorable clinical outcomes than those who received standard medical treatment alone (9, 10). Nevertheless, the rate of recanalization following MT failure remained high (up to 29%), and poor clinical outcomes were demonstrated in these patients. Recanalization is a predictor that is strongly associated with good outcomes (11). Several attempts have been made to improve the efficacy of recanalization in MT using the current tools (12, 13). Some studies have suggested that permanent rescue stenting (RS) may be a promising modality for intracranial large artery occlusion with failed MT (14–16). However, the literature on this topic is mainly focused on the anterior circulation with only a number of small case series on the posterior circulation (13, 17).

Therefore, in the present study, we evaluated the feasibility of intracranial RS in patients with basilar artery occlusion (BAO) with MT failure and hypothesized that permanent stenting may be effective and safe in them.

## Materials and Methods

### Patient Selection

Patients with acute BAO who underwent MT with failed recanalization were identified from the Endovascular Treatment Key Technique and Emergency Work Flow Improvement of Acute Ischemic Stroke (ANGEL-ACT) database (ClinicalTrials.gov Identifier: NCT 03370939). The ANGEL-ACT study prospectively recruited consecutive patients with intracranial large vessel occlusion who underwent endovascular treatment (EVT) at 111 comprehensive stroke centers in China over 2 years, and the main article was published previously (18). The enrollment criteria in the present study were as follows: (1) age ≥ 18 years; (2) initial National Institutes of Health Stroke Scale (NIHSS) score ≥ 10; (3) onset-to-puncture time ≤ 24 h; (4) acute posterior circulation stroke; and (5) BAO documented during EVT.

### Data Collection and Assessment

Data of each patient, including the baseline characteristics, risk factor profile, procedural details, and time metrics, were collected using a pre-defined electronic case report form (eCRF) in the electronic data capture (EDC) system. Some original records of the patients, including the laboratory findings and electrocardiograms, were uploaded to the EDC as photographs. Before enrollment, all sites were uniformly trained regarding the use of the EDC system for data entry and electronic signatures. The EDC system data underwent central quality checks by blinded statisticians to control for consistency, accuracy, and completeness. Data queries were sent in cases of inconsistencies or missing data. All imaging data were copied to optical discs and sent to the central core laboratory, including pre-procedure non-enhanced computed tomography (NECT) with computed tomography angiogram (CTA) and/or diffusion-weighted magnetic resonance imaging (MRI) with magnetic resonance angiography (MRA) and digital subtraction angiography (DSA) during EVT, and follow-up computed tomography (CT) or MRI at 24 ± 6 h post-procedure. CT was performed if a patient's neurological status deteriorated abruptly.

### Radiological Assessment

Baseline CT/MRI and CTA/MRA, DSA, and post-procedural imaging were evaluated by an imaging core laboratory blinded to the clinical data and outcomes. The pre-procedure posterior circulation Alberta stroke program early CT score (pc-ASPECTS) was evaluated using a baseline non-contrast CT. Successful recanalization was defined as an mTICI score of 2b or 3 on the final catheter angiogram. All neuroimaging data were assessed independently by two neuroradiologists who were blinded to the clinical data; in cases of disagreements, decisions were made by a third experienced neuroradiologist.

### EVT

EVT was performed under general anesthesia or conscious sedation at all the participating centers. The type of MT device was at the operator's discretion in each center for the study was a retrospective analysis of the data collected from each stroke center. In cases of failed MT with persistent high-grade stenosis (>70% stenosis according to the Warfarin Aspirin Symptomatic Intracranial Disease criteria) (19) and a tendency toward immediate reocclusion, RS was performed at the discretion of the operator. The antiplatelet and anticoagulant strategies during and/or after the procedure were also dependent on the consensus between the operator and the stroke neurologist according to each hospital's protocol. The protocols mainly followed the following principles. A glycoprotein IIb/IIIa inhibitor (tirofiban) was administered according to the recommendations from the manufacturer; it was initiated with a weight-adapted intravenous loading dose of 0.4 μg/kg/min over 30 min followed by a continuous infusion of 0.1 μg/kg/min over 24 h. Dual antiplatelet therapy with 100 mg aspirin and 75 mg clopidogrel was started either orally or via a nasogastric tube 4 h before stopping the infusion of tirofiban.

### Follow-Up

CT or MRI was performed in all patients within 2 days of EVT as per each center's follow-up protocol. Post-procedural clinical assessment was performed at 3 months *via* telephone from the study's central laboratory, which included assessments of the modified Rankin Scale (mRS) score, Barthel Index (BI) score, and death due to any cause. Radiographic examinations (DSA/MRA/CTA) were repeated 24 ± 6 h after stenting to evaluate the patency of the stent or target vessel.

### Outcomes

The rate of successful recanalization was evaluated in both the RS and non-RS groups. The baseline characteristics, laboratory findings, use of an intravenous tissue plasminogen activator, procedural details, target vessel and stent patency rates at 24 ± 6 h after EVT, rate of good clinical outcomes, the incidence of symptomatic intracranial hemorrhage (sICH), and mortality were compared between the two groups. A favorable outcome was defined as an mRS score of 0–3 at 3 months. A good outcome was defined as an mRS of 0–2 at 3 months. sICH was diagnosed in cases of newly observed intracranial hemorrhage with an increase in NIHSS score by at least 4 points with no other evident causes for this change.

### Statistical Analysis

Statistical analyses were performed using SAS v9.0 (SAS Institute, Cary, NC, USA). Categorical variables are expressed as number (frequency), and quantitative variables are presented as median ± interquartile range or mean ± standard deviation. The baseline demographic characteristics, risk factors, imaging characteristics, tool used for MT, and clinical outcomes were compared between the groups using the χ^2^ test or Fisher exact test for categorical and binary data, respectively, and the Mann–Whitney U test or Student's *t*-test for continuous data. Statistical significance was set at *P* < 0.05.

## Results

### Accrual of Patients

**Figure 1:**
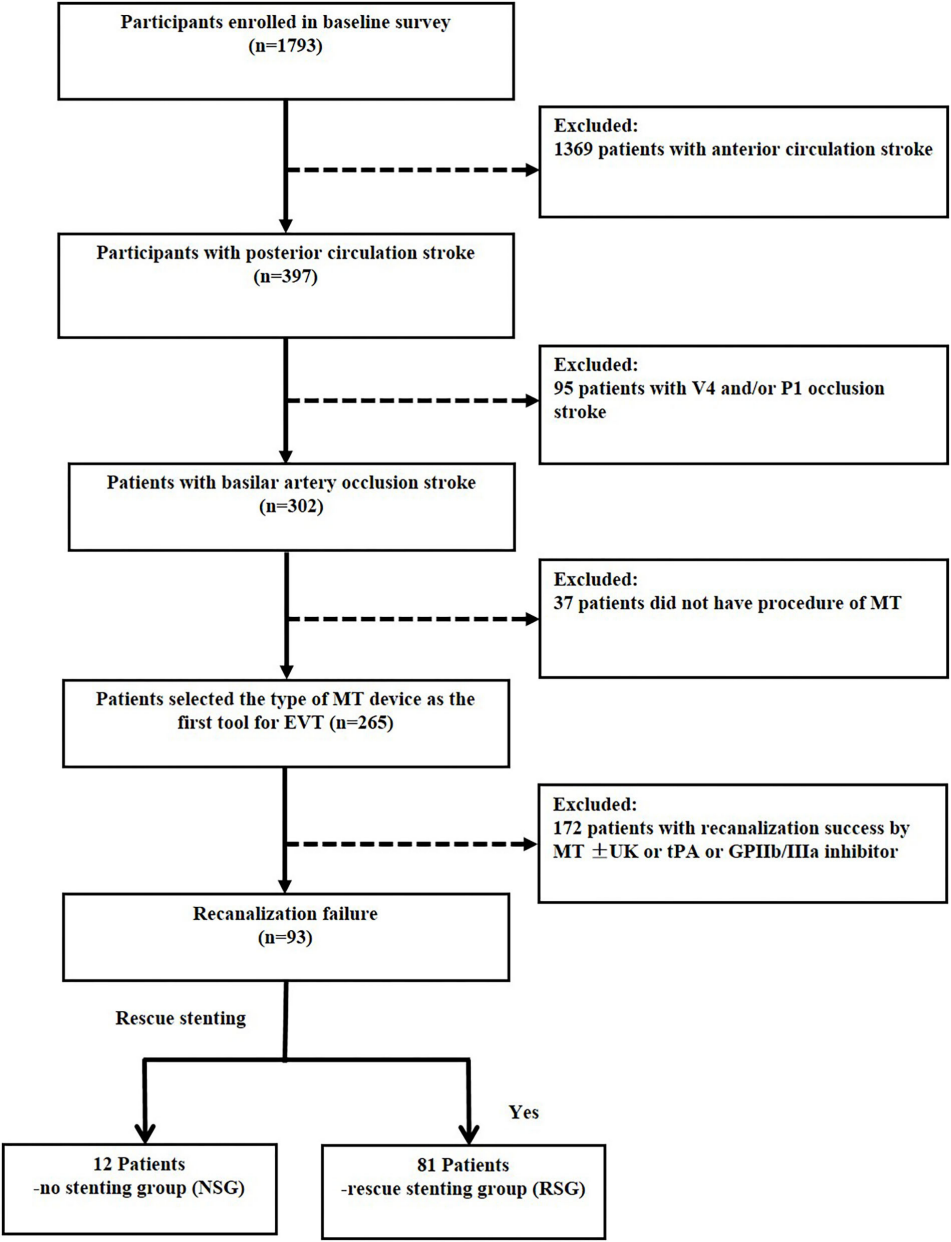
Flow chart of patient inclusion. MT indicates mechanical thrombectomy; V4, forth segment of vertebral artery; P1, first segment of posterior cerebral artery. Recanalization success, modified Tissue Thrombolysis in Cerebral Ischemia, 2b−3.

Overall, 1,793 patients were enrolled from comprehensive stroke centers across China. Of them, 397 had posterior circulation large vessel occlusion. MT failure was identified in 93 patients with BAO who were eligible for inclusion in the current study, and a flowchart depicting the inclusion of patients is presented in Figure 1.

### Baseline Characteristics

Of 93 patients with BAO with failed MT (23.4%), 81 were treated with permanent RS. Recanalization with an mTICI score of 2b-3 was achieved in 92.6% (75/81) of patients in the RS group, while 12 patients did not undergo recanalization with stenting. The baseline demographic data, characteristics, procedures, and clinical outcomes are summarized in Table 1. Overall, the mean age was 61.4 ± 12.0 years and 80 (86%) patients were male. A majority of the patients (64/84, 76.2%) had atherosclerosis as the Trial of Org 10172 in Acute Stroke Treatment (TOAST) subtype, while nine patients had missing data regarding TOAST classification. The mean NIHSS and Glasgow Coma Scale (GCS) scores at admission were 23 (13–25) and 7 (5–11), respectively. Sixteen patients (17.2%) received intravenous thrombolysis in conjunction with MT.

**Table 1:** Comparisons of the clinical, laboratory test, procedural factors and outcomes of acute posterior circulation stroke due to basilar artery occlusion between the rescue stenting and non-stenting groups.

	**Total (*n* = 93)**	**Non-stenting (*n* = 12)**	**Rescue stent (*n* = 81)**	***P*-value**
Age, y; (mean ± SD)	61.4 ± 12.0	63.3 ± 13.4	61.1 ± 11.8	0.709
Male	80 (86.0)	9 (75.0)	71 (87.7)	0.365
**Risk factors**				
Hypertension	61 (65.6)	8 (66.7)	53 (65.4)	>0.999
Diabetes mellitus	15 (16.1)	12 (14.8)	3 (25.0)	0.403
Dyslipidemia	8 (8.6)	1(8.3)	7 (8.6)	>0.999
Smoker	45 (48.4)	5 (41.7)	40 (49.4)	0.562
CHD	16 (17.2)	1 (8.3)	15 (18.5)	0.684
Atrial fibrillation	8 (8.6)	1 (8.3)	6 (7.4)	>0.999
Previous ischemic stroke	20 (21.5)	2 (16.7)	18 (22.2)	>0.999
**Parameters on admission**				
BP systolic (mmHG; median, IQR)	150 (137–165)	150 (142–162)	150 (137–165)	0.805
BP diastolic (mmHG; median, IQR)	90 (85–98)	90 (83–100)	90 (85–98)	0.936
Glucose (mmol/L; mean±SD)	8.3 ± 3.4	8.0 ± 3.0	8.4 ± 3.5	0.721
Platelets (G/l; median, IQR)	226 (195–257)	184 (156–248)	229 (203–257)	0.025
Initial NIHSS, median (range)	23 (13–25)	20 (13–29)	23 (13–35)	0.408
Initial GCS, median (range)	7 (5–11)	7(4–11)	7 (5–11)	0.574
Cause of stroke				0.768
Atherosclerotic	64 (76.2)	6 (66.7)	58 (77.3)	
Cardiac embolism	6 (7.1)	1 (11.1)	5 (6.7)	
Other or unknown	14 (16.7)	2 (22.2)	12 (16.0)	
Tandem lesion	21 (22.6)	2 (16.7)	19 (23.5)	0.728
Onset to admission time, mins; median (range)	120 (35–216)	150 (35–254)	120 (38–203)	0.632
Onset to imaging time, mins; median (range)	125 (46–221)	159 (62–267)	123 (41–220)	0.507
Onset to puncture time, mins; median (range)	260 (200–420)	286 (205–543)	260 (200–415)	0.697
Procedural time mins; median (range)	108 (70–162)	203 (155–229)	104 (67–145)	<0.001
IV tPA	16 (17.2)	4 (33.3)	12 (14.8)	0.211
ASPECT, median (range)	7 (6–8)	7 (6–8)	7 (6–8)	0.800
MT tool used				0.396
Stent riever	85 (90.4)	11 (91.7)	74 (91.4)	
Aspiration	3 (3.2)	1 (8.3)	2 (2.5)	
Both	5 (5.4)	0	5 (6.2)	
No. of MT attempt, mean ± SD	2 ± 2	3 ± 2	2 ± 1	0.003
GPI using	82 (88.2)	8 (66.7)	74 (91.4)	0.033
mTICI 2b-3	75 (80.6)	0	75 (92.6)	<0.001
Balloon angioplasty	47 (50.5)	4 (33.3)	43 (53.1)	0.231
Syptomatic ICH	3 (3.2)	1 (8.3)	1 (1.2)	0.243
mRS 0–3	44 (47.3)	2 (16.7)	42 (51.9)	0.023
BI ≥60	38 (40.9)	1 (8.3)	37 (45.7)	0.014
Mortality	22 (23.7)	7 (58.3)	15 (18.5)	0.006

*CHD, coronary artery disease; GPI, glycoprotein IIb/IIIa inhibitor; IA, intra-arterial; ICA, internal carotid artery; ICH, intracranial hemorrhage; IQR, interquartile range; IV, intravenous; mRS, modified Rankin Scale score; MT, mechanical thrombectomy; mTICI, modified Tissue Thrombolysis in Cerebral Ischemia; NIHSS, National Institutes of Health Stroke Scale; RS, rescue stenting; tPA, tissue-type plasminogen activator; and UK, urokinase; BA, basilar artery*.

### Procedural Characteristics and Complications

Most patients (85/93, 90.4%) were treated using the primary stent retriever technique, while three patients (3.2%) were treated with primary aspiration, and the remaining five patients (5.4%) were treated with both MT devices. The mean number of MT attempts was 2 ± 2, and 82 patients (88.2%) were treated with a glycoprotein IIb/IIIa inhibitor. The median time between the onset of symptoms and final recanalization was 360 min (range, 297–475 min). Complete successful reperfusion (mTICI score: 2b-3) was achieved in 80.6% of patients. sICH was observed in three patients (3.2%).

### Follow Up

After 3 months, 44/93 patients (47.3%) had a favorable clinical outcome (mRS score: 0–3). The functional independence rate was 40.9% (BI score ≥ 60). The all-cause mortality rate at follow-up within 90 days was 23.7% (22/93).

### Baseline and Procedural Characteristics Between Non-rs Group and RS Group

There was no significant difference in the risk factors of stroke between the groups at baseline. The two groups also did not demonstrate differences in the initial NIHSS score, GCS, blood pressure, blood glucose level, pc-ASPECT score, or causes of stroke categorized according to the TOAST classification. Patients in the RS group had a significantly higher platelet count on admission than those in the non-RS group (229 vs. 184 g/L, respectively; *P* = 0.025). The duration between the onset of symptoms and admission (150 vs. 120 min, respectively; *P* = 0.632) and that between the onset of symptoms and puncture (286 vs. 260 min, respectively; *P* = 0.697) were longer in the non-RS group than those in the RS group; however, the differences were not statistically significant. The procedure time was significantly longer in the non-RS group than that in the RS group (203 vs. 104 min, respectively; *P* < 0.001), and the mean number of attempts of MT was higher in the non-RS group than that in the RS group (3 ± 2 vs. 2 ± 1, respectively; *P* = 0.003). However, there were no differences in the overall first-line modality used for MT between the groups. The rate of use of a glycoprotein IIb/IIIa inhibitor was higher in the RS group than that in the non-RS group (91.4 vs. 66.7%, respectively; *P* = 0.033).

### Favorable Outcome and Complications Between Non-rs Group and RS Group

**Figure 2:**
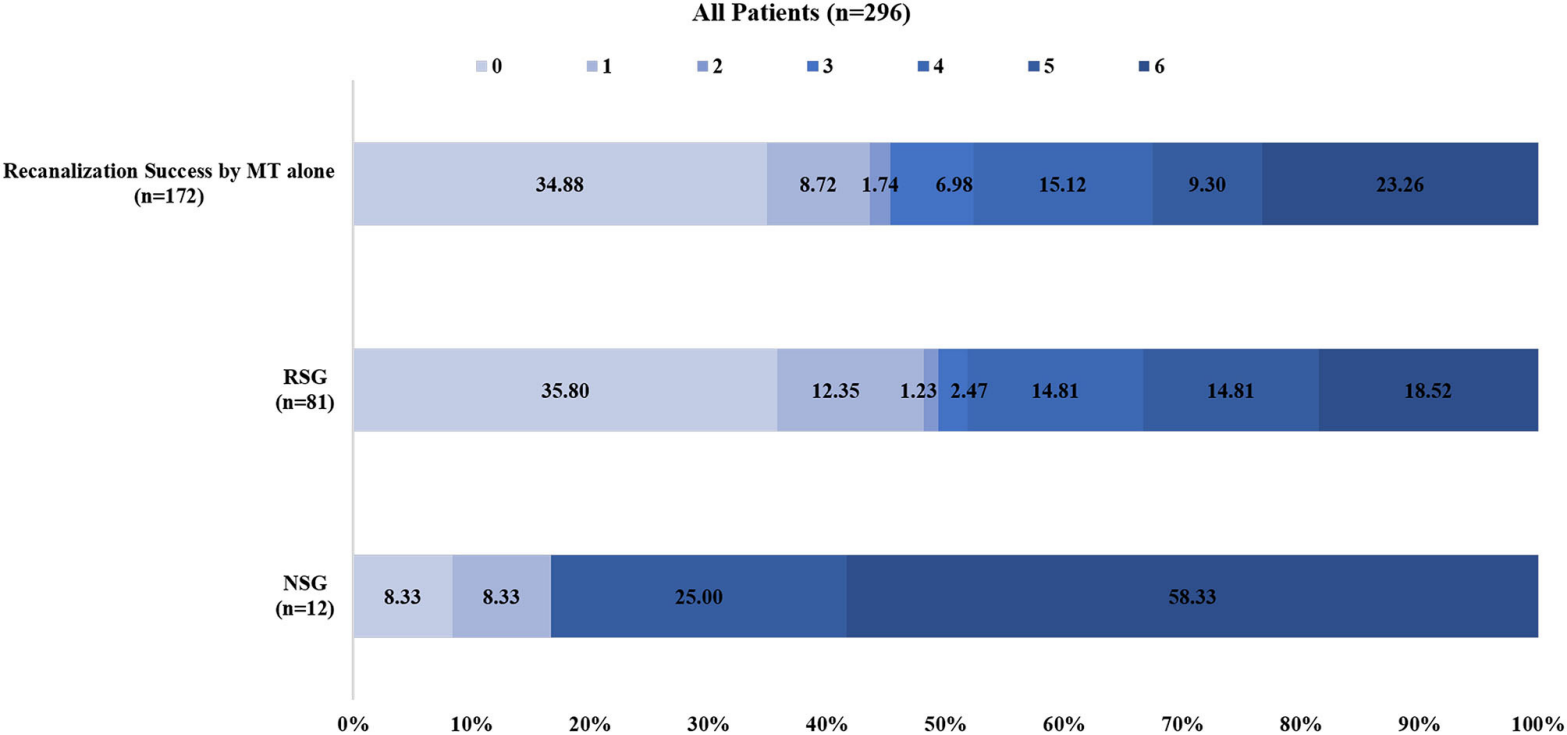
Distributions of the 3-month modified Rankin Scale score (mRS) of patients with recanalization success by mechanical thrombectomy alone and patients with rescue stenting and without rescue stenting after mechanical. MT indicates mechanical thrombectomy; Recanalization success, modified Tissue Thrombolysis in Cerebral Ischemia, 2b−3; RSG, rescue stenting group; NSG, no stenting group.

In the RS group, recanalization with an mTICI score of 2b-3 was achieved in 92.6% (75/81) of patients, and 51.9% (42/81) of patients had a favorable clinical outcome (mRS ≤ 3). Furthermore, of the patients in the RS group with successful recanalization, 52% (39/75) of them had a favorable clinical outcome, which was comparable to the rate of favorable clinical outcomes (52.3%, 90/172) in patients with successful recanalization with MT alone (52 vs. 52.3%, *P* = 0.962) (Figures 1, 2). In contrast, the proportions of favorable outcomes (mRS ≤ 3) and functional independence (BI score ≥ 60) at 90 days after the procedure were lower in the non-RS group than those in the RS group (16.7 vs. 51.9%, *P* = 0.023, and 8.3 vs. 45.7%, *P* = 0.014, respectively). sICH at 24 h was observed in 1.2% of patients in the RS group, which was not significantly different from that (8.3%) in the non-RS group. The non-RS group had a significantly higher rate of all-cause mortality within 90 days of follow-up than the RS group (58.3 vs. 18.5%, respectively; *P* = 0.006).

### Effect of Glycoprotein IIb/IIIa Inhibitor and Anticoagulantst on Stent Patentcy

**Figure 3:**
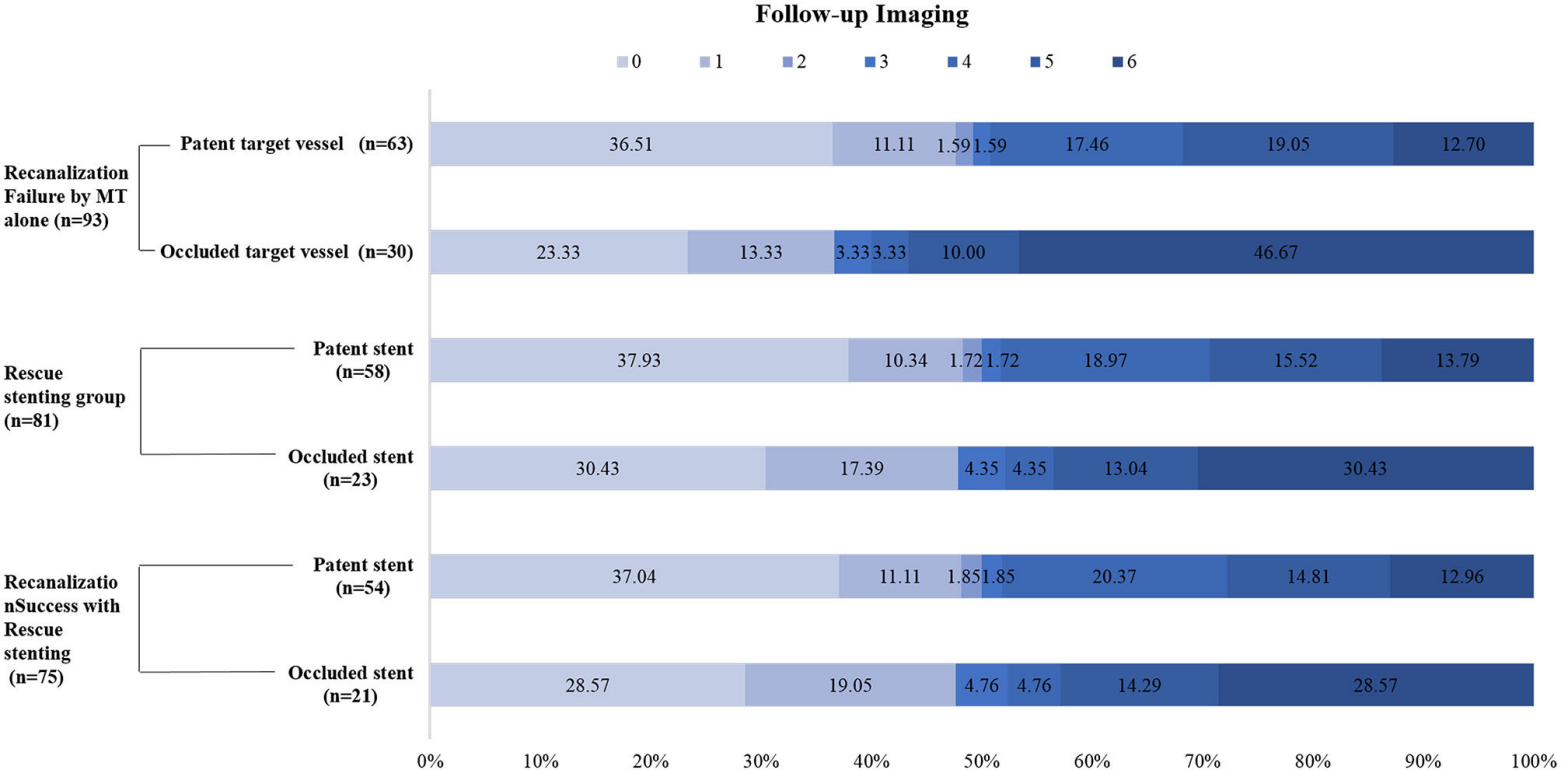
Distributions of the 3-month modified Rankin Scale score (mRS) in MT-failed patients. MT indicates mechanical thrombectomy.

Follow-up imaging at 24 h after stenting revealed that target vessel patency rate was higher in the RS group than that in the non-RS group [71.6% (58/81) vs. 41.7% (5/12), respectively; *P* = 0.038], which was associated with the use of a glycoprotein IIb/IIIa inhibitor (95.2 vs. 73.3%, respectively; *P* = 0.004) during or after the procedure but not with the use of anticoagulants (64.0 vs. 46.0%, respectively; *P* = 0.584) (Table 2). The stent patency rate was higher with the use of glycoprotein IIb/IIIa inhibitors than that without the use of glycoprotein IIb/IIIa inhibitors (94.8 vs. 82.6%, respectively; *P* = 0.096) (Table 3). Neither glycoprotein IIb/IIIa inhibitors nor anticoagulants increased the rate of sICH. Furthermore, compared with patients with occluded target vessels or stents on imaging at 24 h, those with patent vessels or stents had a higher proportion of good clinical outcomes [49.2% (31/63) or 50.0% (29/58)] and a lower rate of mortality [12.7% (8/63) or 13.8% (8/58)], respectively (Figure 3).

**Table 2:** Relationships of glycoprotein IIb/IIIa inhibitor or anticoagulant use to traget vessel patency and symptomatic ICH in MT-failed recanalization patients.

	**Recanalization at 24 h follow-up (*****n*** **=** **93)**			**Symptomatic ICH 24 h follow-up (*****n*** **=** **93)**		
	**Recanalization (*n* = 63)**	**Occlusion (*n* = 30)**	***P*-value**	**Yes (*n* = 2)**	**No (*n* = 91)**	***P*-value**
GPI, *n* (%)			0.004			>0.999
Yes (*n* = 82)	60 (95.2)	22 (73.3)		2 (100)	80 (87.9)	
No (*n* = 11)	3 (4.8)	8 (26.7)		0	11 (12.1)	
Anticoagulant, *n* (%)			0.584			0.192
Yes (*n* = 78)	34 (54.0)	18 (60.0)		0	52 (57.1)	
No (*n* = 41)	29 (46.0)	12 (40.0)		2 (100)	39 (42.9)	

*GPI, glycoprotein IIb/IIIa inhibitor administration during or after the procedure; Anticoagulant, Anticoagulant administration during or after the procedure; ICH, intracranial hemorrhage; RSG, rescue stenting group*.

**Table 3:** Relationships of glycoprotein IIb/IIIa inhibitor or anticoagulant use to patency and symptomatic ICH in rescue stenting group.

	**Recanalization at 24 h follow-up (*****n*** **=** **81)**			**Symptomatic ICH 24 h follow-up (*****n*** **=** **81)**		
	**Recanalization (*n* = 58)**	**Occlusion (*n* = 23)**	***P*-value**	**Yes (*n* = 1)**	**No (*n* = 80)**	***P*-value**
GPI, *n* (%)			0.096			>0.999
Yes (*n* = 74)	55 (94.8)	19 (82.6)		1 (100)	73 (91.3)	
No (*n* = 7)	3 (5.2)	4 (17.4)		0	7 (8.7)	
Anticoagulant, *n* (%)			0.912			0.444
Yes (*n* = 45)	32 (55.2)	13 (56.5)		0	45 (56.2)	
No (*n* = 36)	26 (44.8)	10 (43.5)		1 (100)	35 (43.8)	

*Anti-plt indicates oral antiplatelet drug medication after the procedure, administration of aspirin, clopidogrel, or both; GPI, glycoprotein IIb/IIIa inhibitor administration during or after the procedure; ICH, intracranial hemorrhage; RSG, rescue stenting group*.

## Discussion

We reported the results of RS in patients with acute BAO with failed MT using data collected from the largest prospective registry study (ANGEL-ACT) in China. Our study demonstrated that in patients with acute BAO with MT failure, successful recanalization can be achieved using RS. A higher rate of favorable or functional outcomes was observed in the RS group than that in the non-RS group. Additionally, a trend of lower mortality was observed in the permanent RS group when compared with the non-RS group, which was comparable with the clinical outcome of the successful recanalization initially by MT alone in the present study (Figure 2).

The BEST trial and BASILAR registry study have suggested that patients with acute posterior circulation stroke in whom successful reperfusion was achieved (mTICI score of 2b or 3) had significantly better clinical outcomes than those who received standard medical treatment alone in acute posterior circulation stroke (9, 10). However, failure of reperfusion was reported to be 18–50% with MT alone in a subset of patients with posterior circulation stroke due to various factors (9, 10, 20, 21). In the BASILAR study, the relatively lower successful recanalization rate and poorer outcomes were ascribed to the high rate (65%) of intracranial atherosclerotic stenosis (ICAS) when compared with other recent observational studies (10, 22, 23). Previous studies have also concluded that ICAS is one of the most important factors of MT failure and immediate reocclusion (up to 77%) of the target vessel following MT (13, 17, 24), which was more frequent in the posterior circulation and the Asian population (25). Although the BEST trial did not demonstrate positive results for various reasons (i.e., lower successful recanalization rate 71%, high crossover rate, and high ICAS rate of 56%), patients with successful reperfusion (mTICI score ≥ 2b) had a significantly higher rate of good clinical outcomes than those who received standard medical treatment (9). Recently, the BASICS trail also revealed that the higher rate of patency of the basilar artery at 24 h, the higher incidence of the favorable outcome at 90 days. This observation validates the theory that successful recanalization is one of the most powerful factors for a good outcome. Several studies have demonstrated that RS might be an effective strategy for achieving recanalization in patients with anterior circulation large artery occlusion with failed MT and can result in good clinical outcomes (14–16). This strategy may also be applied to posterior circulation large artery occlusion following MT failure. In our study, ICAS accounted for a high proportion of patients (178/281, 63.4%; 21 patients had missing data regarding the TOAST classification). Additionally, 81 patients (26.8%) underwent RS in this study, which was more than those in the BASILAR study (10.2% of the patients underwent rescue balloon angioplasty and/or stenting). The rates of successful reperfusion and functional independence at 3 months in our study were higher than those in the BASILAR study, the BEST trial and the BASICS trial, and the results were comparable to those of the TREVO registry study and other previously published studies (22, 23, 26). This may be because of numerous factors. First, the successful recanalization rate in the RS group was higher than those in the BASILAR study. The BEST trial and the BASICS trial, which is one of the most important factors for achieving good clinical outcomes following acute ischemic stroke (27, 28). Second, the median procedure time in the RS group was shorter in the current study than those in the BASILAR study and the BEST trial. Furthermore, the number of device passes in the RS group was significantly lower than that in the non-RS group, which could have shortened the time to reperfusion and decreased the infarct volume. Third, RS in patients with failed MT could potentially push the plaque into the smaller perforating branches and cause new ischemia, as reported by the Stenting vs. Aggressive Medical Therapy for Intracranial Arterial Stenosis (SAMMPRIS) trial; however, it is less important in patients with acute stroke because occlusion of the perforators would have already occurred in most cases (16, 29). Forth, in the BASICS trial, although a difference that was not statistically different in the two groups. The second analysis indicated that those with an NIHSS score of ≥10 tended to have better outcomes with EVT, which was included in our enrollment criteria. In addition, fewer device passes might also reduce the rate of complications (30, 31). In terms of the mortality rate, our results support previous findings of lower mortality associated with successful recanalization (32).

Stent reocclusion is one of the major challenges in RS. Glycoprotein IIb/IIIa inhibitors, anticoagulants, or oral antiplatelets are recommended to reduce its risk. In our study, most patients with BAO stroke presented with severe symptoms at admission (coma, locked-in state, or tetraplegia) and could not be prescribed oral antiplatelets; therefore, they were administered glycoprotein IIb/IIIa inhibitors or anticoagulants. Unfortunately, the current study could not determine the best antiplatelet therapy to balance the risk of intracranial hemorrhage and stent reocclusion. Nevertheless, a glycoprotein IIb/IIIa inhibitor could significantly maintain the patency of the occluded vessel following RS in patients with failed MT without increasing the rate of sICH at 24 h after the procedure (Table 2). These findings are in line with those of previous studies (14, 15, 33). In patients with successful recanalization in the RS group (Table 3), despite the fact that glycoprotein IIb/IIIa inhibitors tended to maintain the stent patency, no significant differences were found between those who received glycoprotein IIb/IIIa inhibitors and those who did not. This may be due to the high rate of ICAD-related occlusion of the basilar artery in our study. The plaque within an occluded segment is vulnerable to disruption by MT, and RS might further disrupt the unstable plaque. This leads to platelet aggregation and promotes local thrombus formation, thus resulting in reocclusion of the target vessel (34). Another important factor might be the elevated platelet count on admission in the RS group. Mosimann et al. reported that patients with a high platelet count on admission were at a higher risk of reocclusion after MT because a higher concentration of circulating platelets could easily adhere and form new occlusive thrombi; therefore, aggressive antiplatelet therapy is often required in such cases (34, 35). Furthermore, the use of a glycoprotein IIb/IIIa inhibitor (tirofiban) in our study during and/or after the procedure also depended on each hospital's protocol and the manufacturer's guidelines. The differences in the administration and dosage of a glycoprotein IIb/IIIa inhibitor could not be avoided between the centers.

In conclusion, our study suggested that permanent stenting seemed to be a feasible rescue modality in refractory to MT and might provide functional benefit in patients with acute ischemic stroke due to basilar artery occlusion.

### Limitations

Our study has all limitations that come along with a observational study design. Major limitation is the small number of patients without rescue stenting This may be due to each participating hospital had its own protocols regarding referral of patients for RS following the failure of MT for BAO, and the decision regarding performing RS depended on the interventionists and patient conditions. Therefore, selection bias may have affected the results. Second, the present study only enrolled the patients with admission NIHSS ≥ 10, whether the patients with mild stroke (NIHSS <10) on admission could benefit from the RS following MT failure was needed further investigation. Furthermore, our findings should be interpreted cautiously due to regional differences in the prevalence of ICAS, which is more frequent in Asian populations than the European and North American populations.

## Data Availability Statement

The original contributions presented in the study are included in the article/supplementary material, further inquiries can be directed to the corresponding author/s.

## Ethics Statement

The studies involving human participants were reviewed and approved by Ethical Committee of Beijing Tiantan Hospital. The number of the approved ethical statement is KY2017-048-01. The patients/participants provided their written informed consent to participate in this study.

## Author Contributions

GL and FG: study concept and design and drafted the manuscript. XZ, BJ, XH, RL, MC, and GM: critical revision of manuscript for intellectual content. GP, JZ, ZQ, XG, BH, XT, BW, LS, LL, and ZH: performed the data collection and data analysis. DM, NM, XS, MY, and ZM: study concept and design, analysis and interpretation of data, and critical revision of manuscript for intellectual content. All authors contributed to the article and approved the submitted version.

## Funding

The study was supported by grants from Beijing Hospitals Authority Youth Programme (No. QML20190505) and Ministry of Science and Technology of the People's Republic of China (2016YFC1301501).

## Conflict of Interest

The authors declare that the research was conducted in the absence of any commercial or financial relationships that could be construed as a potential conflict of interest.

## Publisher's Note

All claims expressed in this article are solely those of the authors and do not necessarily represent those of their affiliated organizations, or those of the publisher, the editors and the reviewers. Any product that may be evaluated in this article, or claim that may be made by its manufacturer, is not guaranteed or endorsed by the publisher.
